# Babam2 negatively regulates osteoclastogenesis by interacting with Hey1 to inhibit Nfatc1 transcription

**DOI:** 10.7150/ijbs.72487

**Published:** 2022-07-11

**Authors:** Fujun Jin, Yexuan Zhu, Meijing Liu, Rongze Wang, Yi Cui, Yanting Wu, Gang Liu, Yifei Wang, Xiaogang Wang, Zhe Ren

**Affiliations:** 1 Key Laboratory of Big Data-Based Precision Medicine, School of Engineering Medicine, Beihang University, Beijing 100191, China.; 2 Guangzhou Jinan Biomedicine Research and Development Center, Institute of Biomedicine, College of Life Science and Technology, Jinan University, Guangzhou 510632, China.; 3 Department of Rehabilitation Medicine, Nanfang Hospital, Southern Medical University, Guangzhou 510515, China.

**Keywords:** Bone resorption, Osteoclasts, Babam2, Hey1, Nfatc1, Osteoporosis

## Abstract

Osteoclast-mediated excessive bone resorption was highly related to diverse bone diseases including osteoporosis. BRISC and BRCA1-A complex member 2 (Babam2) was an evolutionarily conserved protein that is highly expressed in bone tissues. However, whether Babam2 is involved in osteoclast formation is still unclear. In this study, we identify Babam2 as an essential negative regulator of osteoclast formation. We demonstrate that Babam2 knockdown significantly accelerated osteoclast formation and activity, while Babam2 overexpression blocked osteoclast formation and activity. Moreover, we demonstrate that the bone resorption activity was significantly downregulated in Babam2-transgenic mice as compared with wild-type littermates. Consistently, the bone mass of the Babam2-transgenic mice was increased. Furthermore, we found that Babam2-transgenic mice were protected from LPS-induced bone resorption activation and thus reduced the calvarial bone lesions. Mechanistically, we demonstrate that the inhibitory effects of Babam2 on osteoclast differentiation were dependent on Hey1. As silencing Hey1 largely diminished the effects of Babam2 on osteoclastogenesis. Finally, we show that Babam2 interacts with Hey1 to inhibit Nfatc1 transcription. In sum, our results suggested that Babam2 negatively regulates osteoclastogenesis and bone resorption by interacting with Hey1 to inhibit Nfatc1 transcription. Therefore, targeting Babam2 may be a novel therapeutic approach for osteoclast-related bone diseases.

## Introduction

Bone homeostasis is maintained by the balance of osteoblast-mediated bone formation and osteoclast-mediated bone resorption processes 1-3. The shift in the balance of these two processes toward bone resorption leads to osteoporosis, a highly prevalent disorder with tremendous harm to the health of elderly people 4-6. Furthermore, the excess activation of bone resorption is also found in many other bone disorders such as invasive bone tumors, rheumatoid arthritis, and periodontal disease 7-10. Therefore, an enhanced understanding of the regulatory mechanism of bone resorption is essential to the fight against bone diseases associated with excessive bone resorption.

Osteoclasts are multinucleated cells differentiated from the bone marrow monocyte-macrophage lineage by the stimulation of macrophage colony-stimulating factor (M-CSF) and receptor activator of nuclear factor-κB ligand (RANKL) 11-13. After binding to the cell surface receptors of osteoclast precursors, M-CSF and RANKL stimulate downstream signaling pathways and activate the transcription of transcription factors essential for osteoclast differentiation and bone resorption activities 14-16. Of these transcription factors, the nuclear factor of activated T cells, cytoplasmic 1 (Nfatc1) is the master regulator for osteoclast formation, which regulates the transcription of various osteoclast marker genes including Ctsk, Trap, Dcstamp, and Mmp9 17-19. The osteoclast-specific Nfatc1 knockout mice display a osteopetrosis phenotype owing to deficiency in osteoclastogenesis 20. Therefore, identifying regulatory molecules and mechanisms underlying the regulation of Nfatc1 in osteoclasts may provide novel insights for the development of antiresorptive strategies 21.

BRISC and BRCA1-A complex member 2 (Babam2) was an evolutionarily conserved protein with diverse cellular functions 22. It is involved in the progression of various types of tumors, including esophageal squamous cell carcinoma, hepatocellular carcinoma, and leukemia 23-25. Babam2 also plays an important role in DNA damage repair, apoptosis, and senescence 26-28. Previous studies demonstrated that Babam2 could enhance the osteoblastic differentiation of the mesenchymal stem cells 29. Nonetheless, the role of Babam2 in osteoclast differentiation has yet to be studied. Moreover, Babam2 has been identified as a modulator of TNF-α, overexpression of Babam2 inhibited TNF-α induced signaling activation 30. As TNF family mediated signaling is implicated in regulating bone resorption 31, 32, it promotes us to explore the role of Babam2 modulation in osteoclast formation.

In this study, we evaluated the effects of Babam2 on osteoclast differentiation in two osteoclastogenesis models in vitro. Through Babam2 knockdown and overexpression experiments, we identify Babam2 as an essential negative regulator of osteoclast differentiation. Furthermore, we validated the role of Babam2 on bone resorption in Babam2-transgenic mice under both physiological and pathological conditions. Mechanistically, we demonstrate that Babam2 interacts with Hey1 to inhibit the transcription of Nfatc1 in osteoclasts. Babam2's negative regulatory activity on osteoclastogenesis indicates that targeting Babam2 may be a novel therapeutic approach for osteoclast-related bone diseases.

## Methods

### Cell culture

RAW264.7 mouse monocyte-macrophages were obtained from the Chinese Academy of Sciences Cell Bank and were maintained in DMEM medium (Gibco, #12100-046) containing 10% FBS (Gibco, #10100-147). To generate osteoclast from RAW264.7 cells, cells were cultured overnight at a density of 20, 000 per milliliter. Then stimulate with 100 ng/ml RANKL (Peprotech, #315-11C) and renewal medium every two days for a total of 3 days 33. Mouse bone marrow-derived macrophages (BMMs) osteoclastogenesis model was constructed following the conventional protocols 34. Briefly, collect the bone marrow from long bones of the C57BL/6J mice, then suspend the bone marrow cells in α-MEM medium (Gibco, #12571-500) containing 10% FBS (Gibco, #10100-147) and culture for one day. Then unattached cells were collected and stimulated with 50 ng/ml M-CSF (Peprotech, #315-02) for one day to generate BMMs. Subsequently, stimulate the BMMs with a osteoclastogenesis induction medium containing 50 ng/ml M-CSF and 100 ng/ml RANKL (Peprotech, #315-11C) and renewal medium every two days for a total of 4 days.

### TRAP staining assay

The TRAP staining assay was performed using a TRAP Staining Kit (Sigma, #387A) according to the manufacturer's instructions. Briefly, the osteoclasts were rinsed once with PBS, fixed for 10 minutes in 4% paraformaldehyde. After that rinse the cells twice with distilled water, and incubate the cells with TRAP staining solution for 1 hour at 37 °C. Then, cells were washed with distilled water to stop the reaction. Pictures were taken using the inverted microscope (Nikon, ECLIPSE Ti-E). The osteoclast number per well was counted by image analysis freeware ImageJ (v.1.52a, NIH).

### Pit formation assay

For pit formation assay, the BMMs or RAW264.7 cells were paled on the Osteo Assay Surface Plate (Corning, #3988) and induced with osteoclastogenesis medium for 6 days. Then, the cell culture media was aspirated and a 10% bleach solution was added to the plate. After incubated with the bleach solution for 5 minutes at room temperature (RT), the plate was washed five times with distilled water and air dry at RT. Osteoclast resorption pits were observed using the inverted microscope (Nikon, ECLIPSE Ti-E). The osteoclast resorption area per well was analyzed by image analysis freeware ImageJ (v.1.52a, NIH).

### Cell transfection

To mediate gene silencing the cells were transfected with specific siRNA SMARTpools targeting Babam2 or Hey1. To mediate gene overexpression the cells were transfected with eukaryote plasmids pcDNA3.1-Babam2 or pcDNA3.1-Hey1. The cell transfection was performed with jetPRIME transfection reagent (Polyplus, #114-15) according to the manufacturer's instructions. Briefly, siRNA or endotoxin-free plasmids were added to jetPRIME transfection buffer and vortex for 10 sec. After that jetPRIME transfection reagent was added to the buffer, vortex for 10 sec, and incubated for 10 min at RT. Finally, added the transfection complex to the plate and incubate for 6 h. Then, exchange medium to induction osteoclast differentiation. The scramble (TTC TCC GAA CGT GTC ACG T), Babam2 siRNA SMARTpool (CAC AGG UGU CGU GGA AUA UTT, UGA AGU UAC CAG UAG AUU UTT, GGU ACA GUA UGU GAU UCA AGG, UCU GGC UGU ACA UCA UUG ATT) and Hey1 siRNA SMARTpool (GCT AGA AAA AGC TGA GAT CTT, CGA CGA GAC CGA ATC AAT TTT, GGA CGA GAA TGG AAA CTT GTT, CCA TCT CAA CAAC TAC GCA TTT) were synthesized by IBSBio (Shanghai, China).

### Dual-luciferase activity assay

To analyze the Nfatc1 promoter activities, BMMs were plated in a 24-well plate and transfected with pcDNA3.1-Babam2, pcDNA3.1-Hey1, or empty pcDNA3.1 vector, pGL4.11[luc2P]-mNfatc1 promoter and pRL-TK for 6 h using jetPRIME transfection reagent. After stimulating the transfected cells with an osteoclastogenesis induction medium for 24 hours, the cells were collected and lysis with PLB buffer. Insoluble cell debris was discarded by centrifugation and the clear supernatant was subjected to dual-luciferase activity assays using the Dual-Luciferase Reporter Assay System (Promega, #E1910) according to the manufacturer's instructions.

### Western Blot assay

The western blot assay was performed following the classic protocols that could be found in the textbook. Briefly, cells were rinsed once with PBS and scraped from dishes in SDS lysis buffer (Beyotime, #P0013G) containing protease inhibitors (Beyotime, #P1005), and lytic at 100 °C for 10 min. The soluble supernatant was collected by centrifugation, and the protein concentrations were measured by BCA Protein Assay Kit (Beyotime, #P0012S) and normalized. Proteins were separated by 10% SDS-PAGE and subsequently transferred to PVDF membranes (Millipore, IPVH0010). Then the membranes were blocked with 5% w/v skim milk for 1 h at RT. After that, the membranes were washed three times with TBST buffer and incubated with primary antibodies overnight at 4 °C. Subsequently, the membranes were washed three times with TBST buffer and then incubated with HRP-conjugated secondary antibodies at RT for 1 h. Lastly, the blots were visualized by chemiluminescence reagent (Millipore, #WBLUR0500). The primary antibodies included anti-Hey1 (Proteintech, #19929-1-AP, 1:500), anti-Ctsk (Proteintech, #11239-1-AP, 1:1000), anti-Babam2 (CST, #12457, 1:1000), anti-Hsp90 (CST, #4877, 1:1000), anti-Gapdh (CST, #5174, 1:1000), anti-HA (CST, #3724, 1:1000), anti-FLAG (CST, #14793, 1:1000), anti-p-p65 (CST, #3033, 1:1000), anti-p65 (CST, #8242, 1:1000), anti-p-Akt (CST, #4060, 1:1000), anti-Akt (CST, #9272, 1:1000), anti-p-Erk1/2 (CST, #4370, 1:1000), anti-Erk1/2 (CST, #4695, 1:1000), anti-p-p38 (CST, #4511, 1:1000), anti-p38 (CST, #9212, 1:1000), anti-p-Jnk (CST, #9255, 1:1000), anti-Jnk (CST, #9252, 1:1000), anti-Mmp9 (Abcam, #ab38898, 1:5000), anti-Nfatc1 (Santa, #sc-7294, 1:200).

### Quantitative real-time PCR assay (Q-PCR)

Total RNA was isolated from cells using TRIzol Reagent (Invitrogen, #15596-018) according to the manufacturer's instructions. RNAprep Pure Tissue Kit (Tiangen, #DP431) was used for total RNA isolation from liquid nitrogen homogenized bone tissues. The cDNA was synthesized following the protocols of the PrimeScript RT Reagent Kit with gDNA Eraser Kit (Takara, #RR047A). The real-time PCR analysis mixture was prepared using a TB Green Premix Ex Taq II Kit (Takara, #RR820A) according to the manufacturer's instructions. The gene expression levels were normalized with an internal housekeeping gene Gapdh. Gene-specific primer sequences were listed below, *Gapdh* (F: AGG TCG GTG TGA ACG GAT TTG, R: TGT AGA CCA TGT AGT TGA GGT CA), *Babam2* (F: GTG GGA CTG GAT GCT ACA AA, R: GTA CGG GAT GTG CAG TTT GA), *Nfatc1* (F: ACG CTA CAG CTG TTC ATT GG, R: CTT TGG TGT TGG ACA GGA TG), *Ctsk* (F: GAA GAA GAC TCA CCA GAA GCA G, R: TCC AGG TTA TGG GCA GAG ATT), *Mmp9* (F: GCG GCC CTC AAA GAT GAA CGG, R: GCT GAC TAC GAT AAG GAC GGC A), *Trap* (F: ACG GCT ACT TGC GGT TTC A, R: TCC TTG GGA GGC TGG TCT T).

### Co-immunoprecipitation assay

BMMs were cultured in 100 mm cell culture dishes to 90% confluence. The cells were rinsed once with ice-cold PBS and the total proteins were extracted by 1 ml IP Cell lysis buffer for Western and IP (Beyotime, #P0013). The soluble supernatant was collected by centrifugation, and 1 μg primary antibody was added to the cell lysate. After incubation at 4 °C overnight with gentle rotation, Protein A/G agarose (Santa, #sc-2003) was used to pull down the antibody-protein complexes. After washing the complex four times with lysis buffer, the immunoprecipitated proteins were separated by suspending the agarose in SDS loading buffer and boiling for 5 minutes. For co-immunoprecipitation-MS (Co-IP-MS) assay the purified proteins were separated by SDS-PAGE and the gels were digested with trypsin, and the peptide fragments were identified by LC/MS/MS (Q Exactive, Thermo Scientific).

### Generation of Babam2 transgenic mice

The Babam2-transgenic mice (Babam2-TG) on a C57BL/6J genetic background were generated by Cyagen Biosciences (Guangzhou, China). Briefly, the full-length Babam2 coding sequence was cloned into the pPB[Exp]-CAG plasmid. The linearized pPB[Exp]-CAG>Babam2 plasmid was then microinjected into C57BL/6J oocytes. Next, the oocytes were transferred into pseudopregnant C57BL/6J mice. Finally, the line >5 folds overexpression of Babam2 was identified as Babam2 transgenic mice. The genotyping PCR primers are F: 5′- GCA GCT TTC CTC AGT CAC TTT G -3′, R: 5′- GAA TAA GGA ATG GAC AGC AGG -3′, product size 712 bp. Mouse handling and experimental protocols were approved by the Experiment Animal Care Committee and the Ethics Committee of Jinan University (Guangzhou, China).

### Micro-CT analysis

Mice femurs were scanned by Micro-CT (μCT 50, Scanco Medical, Switzerland). The scanning resolution was set as 14.8 μm for one layer. The threshold value of Micro-CT was set from 212 to 1000. The Micro-CT image data were reconstructed and analyzed using Mimics software (v.13.0, Materialise NV). The femur trabecular bone mass analysis was limited to 100 slices upon the distal femur growth plate. The reconstruction 3D image of the femur was taken with the morphology of the distal growth plate as a reference.

### LPS-induced calvarial lesion model

Eight-week-old male mice were injected subcutaneously above the calvaria with PBS or 25 mg/kg/body weight LPS 17. After 7 days, mice were sacrificed and calvaria bones were collected and analyzed by Micro-CT followed by whole-mount TRAP staining and paraffin section TRAP staining. The TRAP-positive area and osteoclast number were analyzed as described above.

### Statistical analysis

All experimental data are presented as the mean ± SD. Unpaired Student's t-test (two-tailed) was used to determine the statistical differences between the two groups. One-way ANOVA with Tukey's multiple comparisons test or two-way ANOVA with Sidak's multiple comparisons test was used to determine the statistical differences between multiple groups. *P*<0.05 was considered statistically significant. * *P*<0.05, ** *P*<0.01, ***P<0.001, ****P<0.0001. GraphPad Prism version 8.0 was used for all statistical analyses.

## Results

### Babam2 expression was down-regulated during osteoclast differentiation

**Figure 1:**
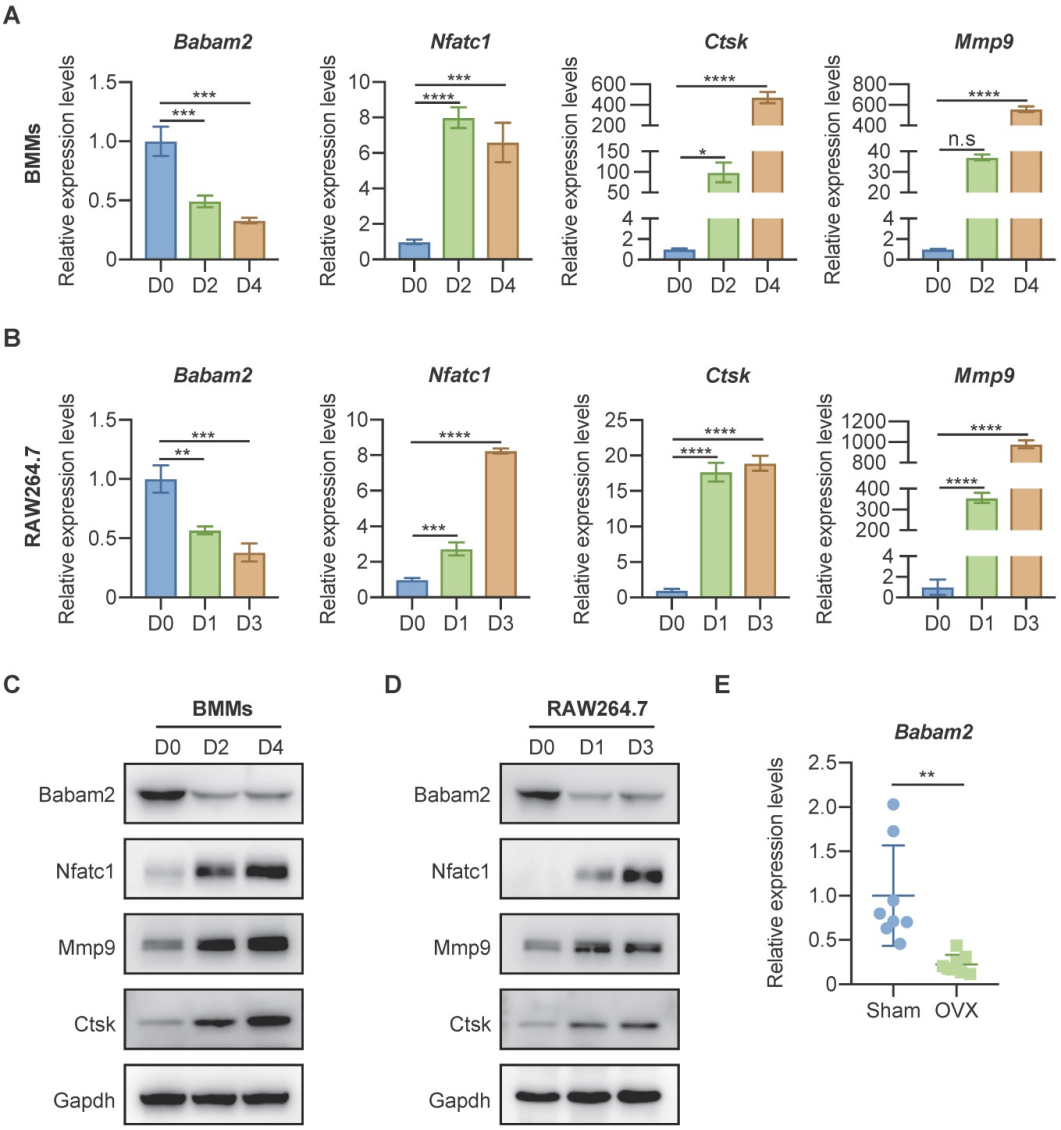
** Babam2 was downregulated during osteoclast differentiation.** (A-B) Q-PCR analysis of *Babam2*, *Nfatc1*, *Ctsk,* and *Mmp9* mRNA expression levels during BMMs (A) and RAW264.7 (B) osteoclast differentiation (n=3). (C-D) Western-blot analysis of Babam2, Nfatc1, Ctsk, and Mmp9 protein expression levels during BMMs (C) and RAW264.7 (D) osteoclast differentiation. (E) Q-PCR analysis of bone tissue *Babam2* gene expression levels from sham and OVX mice (n=8). Data are presented as the means ± S.D. n.s, not significant, *P<0.05, **P<0.01, ***P<0.001, ****P<0.0001.

To investigate whether Babam2 is involved in osteoclast differentiation, we analyzed the expression of Babam2 in mouse bone marrow-derived macrophages (BMMs) osteoclastogenesis models 34 and RAW264.7 mouse monocyte-macrophages osteoclastogenesis models 33. Firstly, total RNA was extracted from osteoclast at different differentiation stages for the quantitative real-time PCR (Q-PCR) gene expression analysis. The results showed that the osteoclast differentiation marker genes, including *Nfatc1*, *Ctsk,* and *Mmp9* were significantly increased during the differentiation processes, indicating the osteoclast differentiation model was constructed successfully (Fig. 1A-B). Interestingly, we found that the gene expression of *Babam2* was significantly reduced during BMMs and RAW264.7 osteoclast differentiation (Fig. 1A-B). Next, we evaluated the changes of Babam2 protein expression in BMMs and RAW264.7 osteoclastogenesis models (Fig. 1C-D). Consistently with the results of mRNA levels, we found that the protein expression of Babam2 also gradually decreased over time (Fig. 1C-D). These results indicate that Babam2 is potentially involved in the osteoclast differentiation processes. Then, we analyzed the *Babam2* gene expression in bone tissues of OVX mice, which is a typical experimental model with enhanced osteoclastogenesis 35. The results showed that the *Babam2* expression levels were significantly decreased in bone tissues of OVX mice compared with sham-operated mice (Fig. 1E). Together, these results indicate that Babam2 was a candidate regulator of osteoclast differentiation, motivating the use of gene loss-of-function experiments to characterize the Babam2 functions further.

### Silencing Babam2 expression promotes osteoclast formation

**Figure 2:**
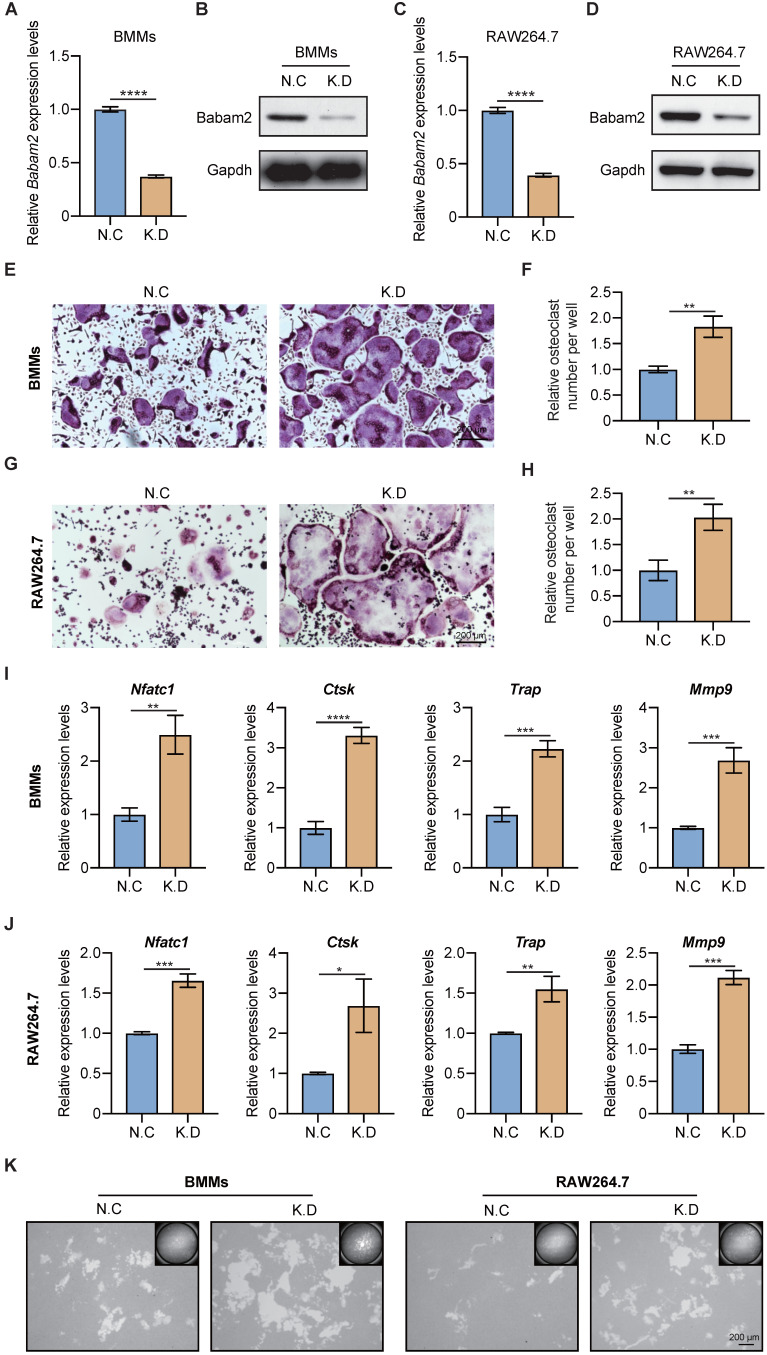
** Babam2 deficiency stimulates osteoclast differentiation.** (A) Q-PCR (n=3) and (B) Western-blot analysis of Babam2 gene knockdown efficiency in BMMs cells. (C) Q-PCR (n=3) and (D) Western-blot analysis of Babam2 gene knockdown efficiency in RAW264.7 cells. (E, G) Representative TRAP staining images and (F, H) quantification of relative osteoclast number per well of the scramble and Babam2 knockdown osteoclasts. (I-J) Q-PCR analysis of *Nfatc1*, *Ctsk, Trap,* and *Mmp9* mRNA expression levels of the scramble and Babam2 knockdown osteoclasts (n=3). (K) Representative images of the osteoclast resorption pits formed by the scramble and Babam2 knockdown osteoclasts in Corning Osteo Assay Surface Plate. Data are presented as the means ± S.D. *P<0.05, **P<0.01, ***P<0.001, ****P<0.0001.

Next, the siRNA SMARTpool targeting Babam2 was transfected into BMMs and RAW264.7 to mediate Babam2 gene silencing. The mRNA and protein expression of Babam2 were significantly downregulated in siRNA transfection groups (K.D) compared with the scramble siRNA transfection groups (N.C), which indicated that siRNA could successfully mediate knockdown of Babam2 expression in BMMs and RAW264.7 cells (Fig. 2A-D). Then, TRAP staining was used to evaluate the effects of Babam2 silencing on osteoclast differentiation. We found that knockdown Babam2 expression significantly stimulated the formation of osteoclasts in both BMMs and RAW264.7 models (Fig. 2E, G). Quantification data showed that Babam2 silencing drastically increased TRAP+ multinucleate osteoclasts numbers (Fig. 2F, H). Furthermore, the Q-PCR results showed that knockdown Babam2 significantly increased the expression of osteoclast differentiation marker genes, including *Nfatc1*, *Ctsk*, *Trap,* and *Mmp9* (Fig. 2I-J). These results demonstrate that the deficiency of Babam2 enhanced osteoclastogenesis. We next sought to determine the role of Babam2 in osteoclast activity. The results of the pit formation assay demonstrate that compared with the N.C osteoclasts the bone resorption area was significantly higher in Babam2 K.D osteoclasts in both BMMs and RAW264.7 models (Fig. 2K), indicating that Babam2 plays an important role in regulating osteoclast bone resorption activity. Taken together, these results indicated that silencing Babam2 promotes osteoclast formation.

### Overexpression Babam2 inhibits osteoclast differentiation

**Figure 3:**
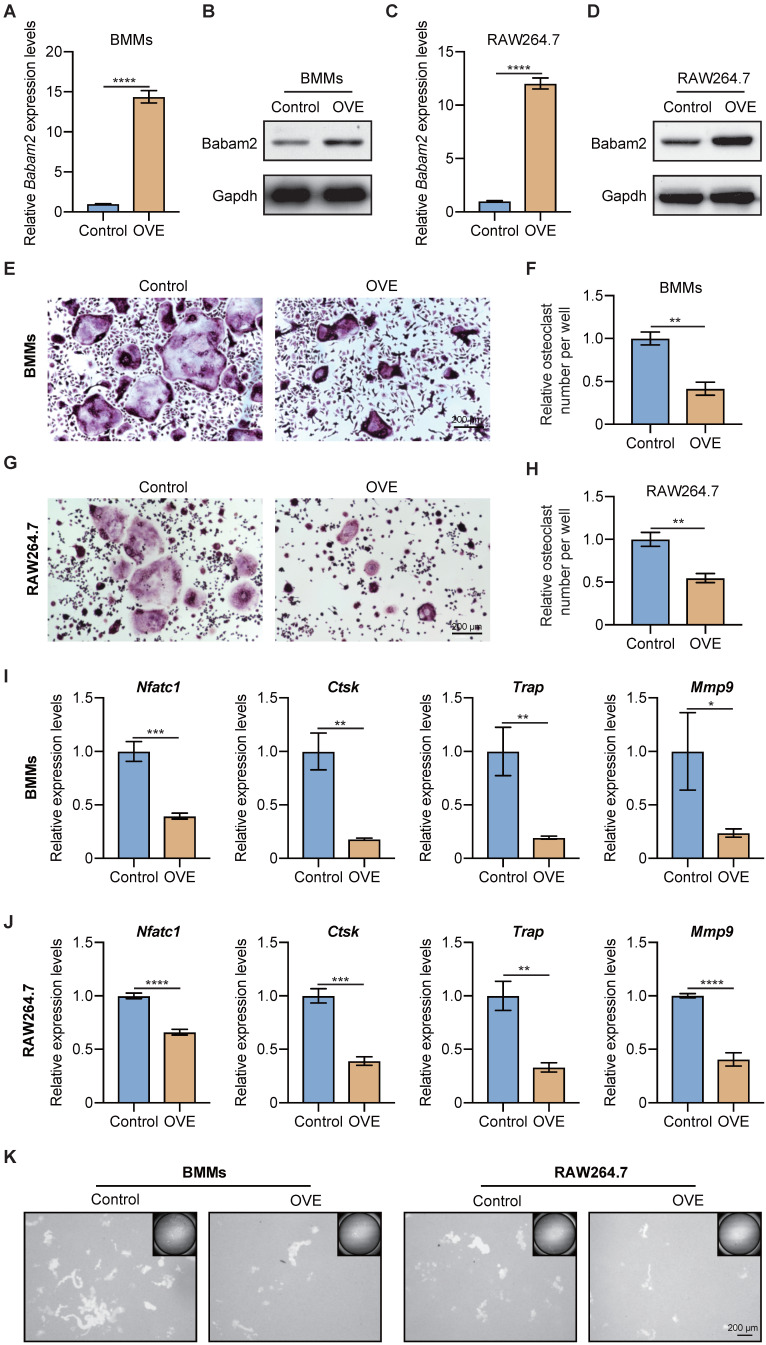
** Babam2 overexpression inhibits osteoclast formation.** (A) Q-PCR (n=3) and (B) Western-blot analysis of Babam2 gene overexpression efficiency in BMMs cells. (C) Q-PCR (n=3) and (D) Western-blot analysis of Babam2 gene overexpression efficiency in RAW264.7 cells. (E, G) Representative TRAP staining images and (F, H) quantification of relative osteoclast number per well of the control and Babam2 overexpressed osteoclasts. (I-J) Q-PCR analysis of *Nfatc1*, *Ctsk, Trap,* and *Mmp9* mRNA expression levels of the control and Babam2 overexpressed osteoclasts (n=3). (K) Representative images of the osteoclast resorption pits formed by the control and Babam2 overexpressed osteoclasts in Corning Osteo Assay Surface Plate. Data are presented as the means ± S.D. *P<0.05, **P<0.01, ***P<0.001, ****P<0.0001.

Next, to further demonstrate the role of Babam2 on osteoclast differentiation, we investigated whether overexpressing Babam2 could inhibit osteoclastogenesis. We overexpressed Babam2 through transfection pcDNA3.1-Babam2 plasmid in BMMs and RAW264.7 cells (Fig. 3A-D). We detected the overexpression efficiency of Babam2 through Q-PCR and western blot assay and the results showed that the mRNA and protein expression of Babam2 were greatly increased in pcDNA3.1-Babam2 plasmid transfection cells (OVE) than in control vector transfection cells (Control) (Fig. 3A-D). Then, we performed TRAP staining and found that Babam2 overexpression significantly inhibited the formation of osteoclasts in both BMMs and RAW264.7 models (Fig. 3E, G). Consistently, quantification data showed that Babam2 overexpression greatly decreased TRAP-positive multinucleated osteoclast numbers (Fig. 3F, H). Meanwhile, we found that overexpression of Babam2 significantly inhibits the expression of osteoclast differentiation marker genes, including *Nfatc1*, *Ctsk, Trap,* and *Mmp9* (Fig. 3I-J). Through pit formation assay, we found that the bone resorption area formed by Babam2 overexpressed osteoclasts was greatly lower than that of control osteoclasts in both BMMs and RAW264.7 models (Fig. 3K). In addition, to determine whether Babam2 can directly affect bone resorption activity, we conducted the Babam2 overexpression and knockdown experiments at the late stage of osteoclast differentiation and evaluated the bone resorption activity by pit formation assay. We found that manipulating the expression of the Babam2 gene in the late stage of osteoclast differentiation only slightly influenced the bone resorption activity of osteoclasts (Fig. S1). Therefore, these results established the negative role of Babam2 on osteoclast differentiation.

### Babam2 transgenic mice show decreased bone resorption

**Figure 4:**
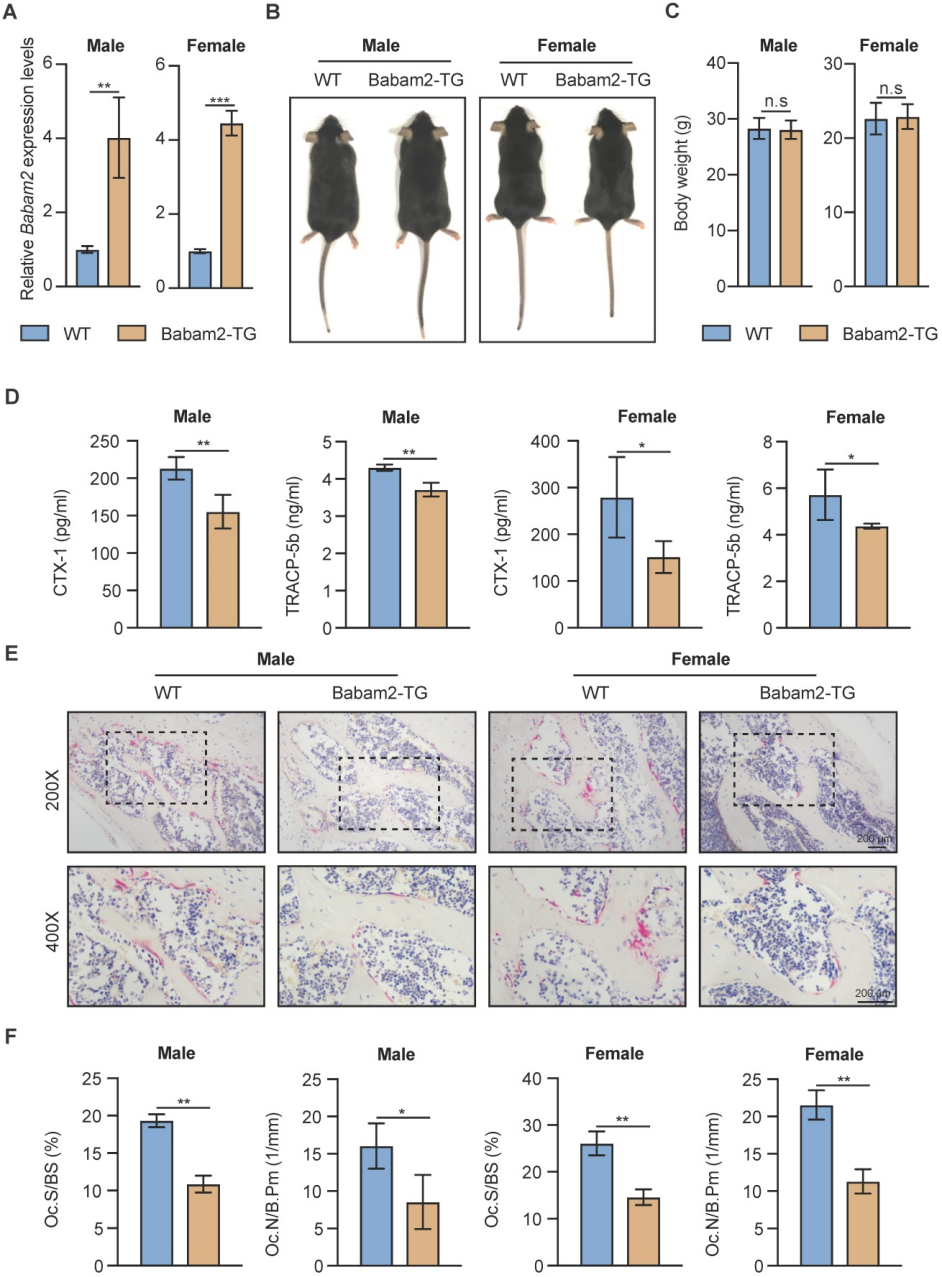
** Babam2 overexpression inhibits bone resorption in mice.** (A) Q-PCR analysis of bone tissue Babam2 gene expression levels in 3-month-old WT and Babam2-TG mice (n=3). (B) General images of 3-month-old WT and Babam2-TG mice. (C) Body weight of the 3-month-old WT and Babam2-TG mice (n=4). (D) Serum bone resorption biomarker CTX-I and TRACP-5b levels of the 3-month-old WT and Babam2-TG mice analyzed by ELISA (n=4). (E) Representative TRAP staining images of femurs from the 3-month-old WT and Babam2-TG mice. (F) Histological osteoclastic metrics Oc.S/BS and Oc.N/B.Pm in subepiphyseal region of femurs of 3-month-old WT and Babam2-TG mice (n=4). Data are presented as the means ± S.D. n.s not significant, *P<0.05, **P<0.01, ***P<0.001.

Next, to explore whether Babam2 could modulate bone resorption in vivo, we generated Babam2 transgenic mice (Babam2-TG). The expression levels of Babam2 were significantly increased in Babam2-TG mice, indicating this model was constructed successfully (Fig. 4A). We found that Babam2 overexpression didn't influence the development of the mice, as indicated by the similar body length and body weight between Babam2-TG mice and the wild-type (WT) littermates (Fig. 4B-C). We performed in vitro osteoclastogenesis assay of cells from Babam2-TG and WT mice to evaluate the effects of Babam2 on osteoclast differentiation. The results showed that the osteoclast differentiation was significantly decreased in cells from Babam2-TG mice as compared with WT littermates (Fig. S2). Therefore, these results further indicated that Babam2 is an essential negative regulator of osteoclastogenesis. Next, we evaluated the bone resorption activities of the Babam2-TG mice. Decreased levels of serum bone resorption biomarkers CTX-I and TRACP-5b indicated that Babam2 overexpression inhibits the bone resorption activities of Babam2-TG mice (Fig. 4D). Then, we directly determined the number of osteoclasts in the femurs of Babam2-TG mice and WT mice by TRAP staining (Fig. 4E). Quantification analysis of the osteoclastic parameters Oc.S/BS and Oc.N/B.Pm showed that the osteoclast formation was significantly decreased in Babam2-TG mice (Fig. 4F). Taken together, these results demonstrated that Babam2 is a negative regulator of bone resorption in vivo. In addition, considering that our previous work indicated that Babam2 could enhance osteoblastic differentiation, we analyzed the osteoblast number of the Babam2-TG mice and the WT littermates. Babam2-TG mice showed an increased number of osteocalcin (OCN)-positive osteoblasts in the bone surface when compared with their WT littermates as detected by immunofluorescence staining (Fig. S3). Therefore, these findings suggest that Babam2 is an essential regulator of bone homeostasis.

### Increased bone mass of Babam2 transgenic mice

**Figure 5:**
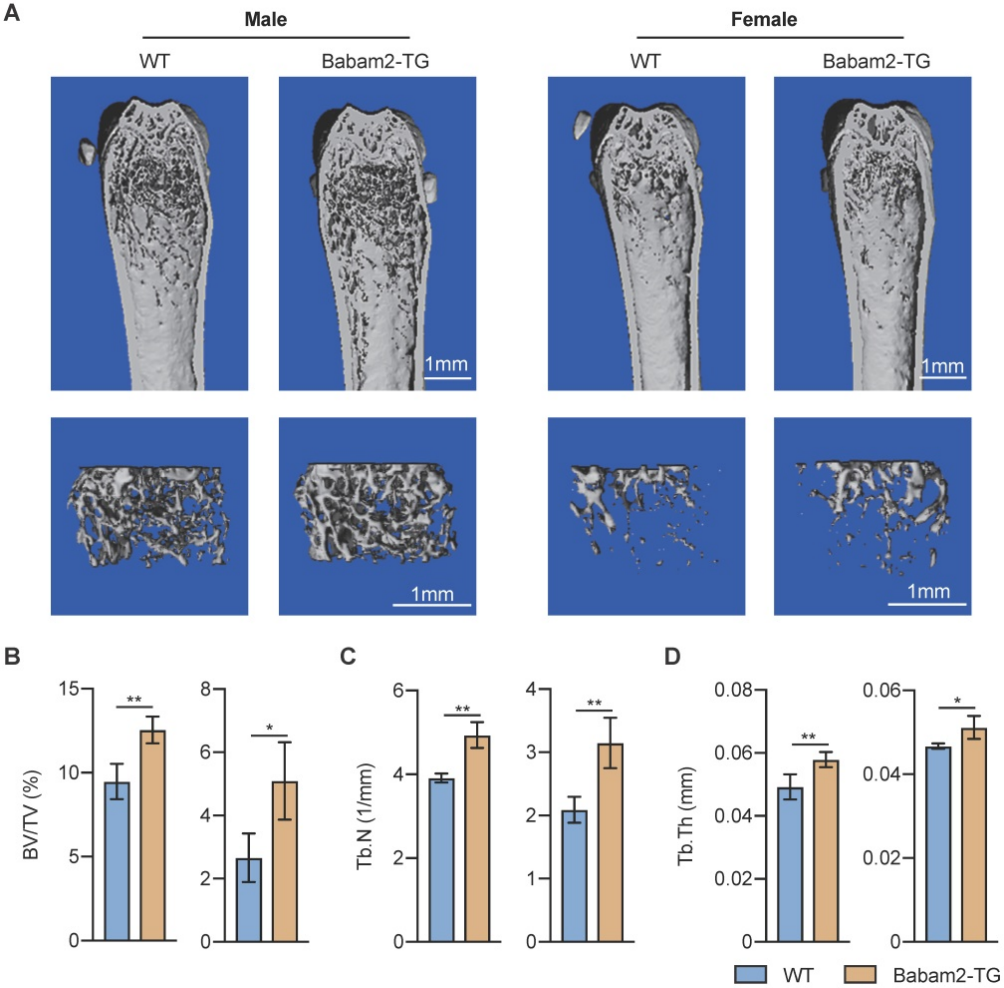
** Increased bone mass of Babam2-transgenic mice.** (A) Representative Micro-CT images showing the 3D bone structures of the femurs from 3-month-old WT and Babam2-TG mice. (B-D) Micro-CT measurements of BV/TV, Tb.N, and Tb.Th in femurs from 3-month-old WT and Babam2-TG mice (n=4). Data are presented as the means ± S.D. *P<0.05, **P<0.01.

Then, we want to know whether Babam2 overexpression will influence the bone mass of Babam2-TG mice. The results of quantitative microtomography (μCT) analysis of the femurs from Babam2-TG mice showed that trabecular bone mass was significantly increased as compared to WT littermates (Fig. 5A). In addition, quantification of the bone parameters including BV/TV, Tb.N, and Tb.Th all greatly increased in Babam2-TG mice (Fig. 5B-D). Thus, these results indicate that overexpressing Babam2 could enhance the bone mass of the mice in vivo.

### Babam2-TG mice are protected from LPS-induced calvarial lesion

**Figure 6:**
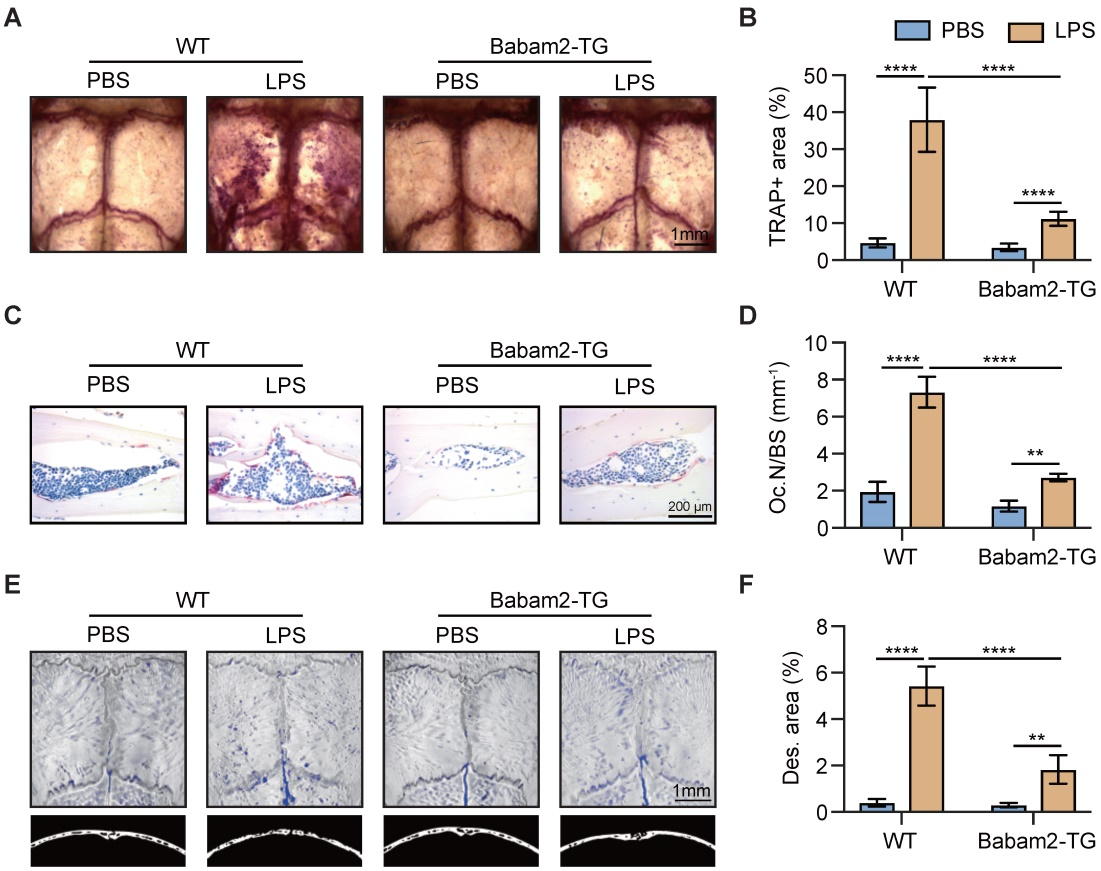
** Babam2-TG mice are protected from LPS-activated bone resorption.** (A) Representative TRAP staining images of whole calvaria bones. (B) Quantification of percentages of TRAP+ area in calvaria bones (n=4). (C) Representative images of TRAP staining of calvarial sections. (D) Quantification of osteoclast number in calvarial TRAP staining sections (n=4). (E) Representative Micro-CT images of mouse calvaria bones. (F) Micro-CT measurements of percentages of bone destruction area in calvaria bones (n=4). Data are presented as the means ± S.D. **P<0.01, ****P<0.0001.

Next, to explore the function of Babam2 in pathologic bone resorption activation-induced bone loss, we constructed a LPS-induced calvarial bone lesion model, which mimics bacteria-induced bone loss. TRAP staining revealed that LPS injections drastically increased the TRAP-positive area in WT calvaria, while such increases were largely abolished in Babam2-TG mice (Fig. 6A-B). Consistently, TRAP staining of the calvarias section showed that the LPS-induced increases of the osteoclast number were greatly inhibited in Babam2-TG mice as compared with WT mice (Fig. 6C-D). Furthermore, the MicroCT analysis data showed that LPS-induced a drastic increase of bone destruction in WT calvaria but not in Babam2-TG calvaria (Figure 6E-F). Taken together, these results indicated that Babam2 contributes to pathologic bone resorption activation and overexpression of Babam2 is a potential therapeutic approach for pathologic bone loss.

### Babam2 binding with Hey1

**Figure 7:**
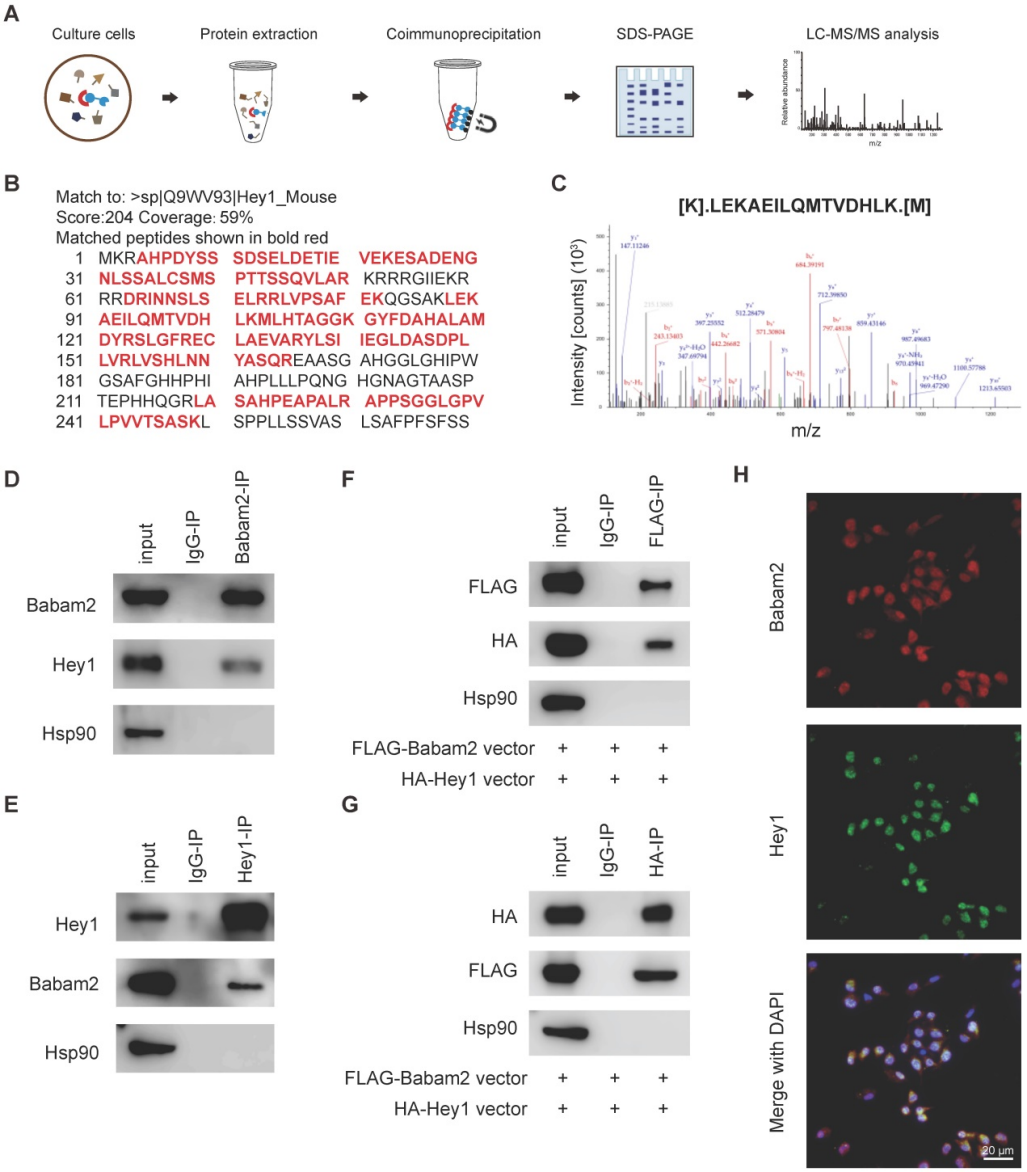
** Babam2 interacts with Hey1.** (A) Schematic diagram of the co-immunoprecipitation-MS (Co-IP-MS) assay. (B-C) Mass spectrometry analysis identified Hey1 as a binding partner of Babam2. (D-E) Representative western blots of the immunoprecipitation assays were performed in BMMs with antibodies specific for Babam2 and Hey1. (F-G) The interaction of the extraneous overexpressed Babam2 and Hey1 was determined by immunoprecipitation assay in 293T cells transfected with pFLAG-Babam2 and pHA-Hey1 plasmids. (H) Representative images of Babam2 (red) and Hey1 (green) immunostaining in BMMs cells.

We next sought to investigate the molecular mechanism through which Babam2 regulates osteoclastogenesis. We performed a co-immunoprecipitation-MS (Co-IP-MS) assay to identify the binding partners of Babam2 (Fig. 7A). Interestingly, Hey1, a negative regulator of osteoclast differentiation was identified as an interaction target of Babam2 in BMMs (Fig. 7B-C). The coverage of the identified peptides reached 59% of Hey1 indicating high interaction properties between Hey1 and Babam2 (Fig. 7B). Then, we verified the interaction between Babam2 and Hey1 with immunoblotting. The results showed that the antibody of Babam2 could co-immunoprecipitation Hey1, meanwhile, the antibody of Hey1 also could co-immunoprecipitation Babam2 (Fig. 7D-E). Accordingly, interaction between Hey1 and Babam2 could be verified by extraneously overexpressing proteins (Fig. 7F-G). Moreover, the results of the immunofluorescence assay showed that Babam2 and Hey1 display strong co-localization in the nucleus of the BMMs cells (Fig. 7H). Therefore, these results demonstrate that Hey1 was a binding partner of Babam2.

### Babam2 cooperates with Hey1 to inhibit Nfatc1 transcription

**Figure 8:**
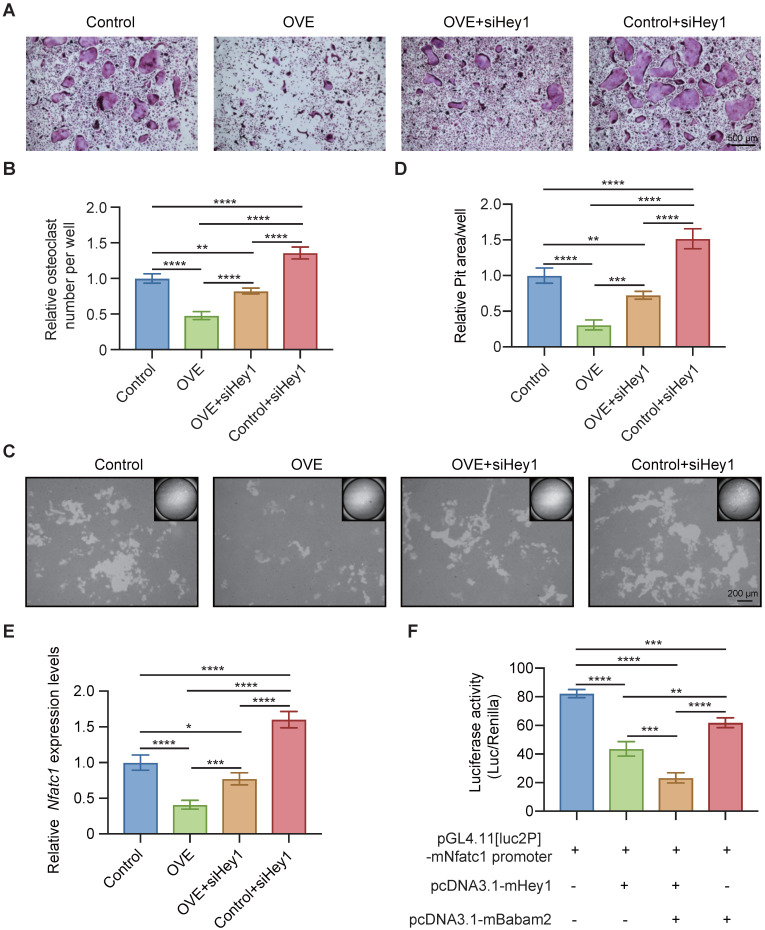
** Babam2 interacts with Hey1 to inhibit Nfatc1 transcription.** (A) Representative TRAP staining images of the osteoclasts were generated in different groups. (B) Quantification of the relative osteoclast number per well of the osteoclasts in different groups (n=3). (C) Representative images of the osteoclast resorption pits in Corning Osteo Assay Surface Plate in different groups. (D) Quantification of the relative osteoclast resorption pit area per well in different groups (n=3). (E) Q-PCR analysis of Nfatc1 gene expression levels of the osteoclasts in different groups (n=3). (F) Nfatc1 promoter activity in different groups was analyzed by dual-luciferase reporter assay (n=3). Data are presented as the means ± S.D. ***P<0.001, ****P<0.0001.

Finally, we used siRNAs to silence Hey1 expression in BMMs to determine whether the Babam2's osteoclastogenesis inhibition activity is mediated by Hey1. We found that the Hey1 knockdown cells formed more osteoclasts and silencing Hey1 largely rescued Babam2 overexpression-induced osteoclastogenesis deficiency (Fig. 8A-B). Consistently, the pit-formation assay also indicated that Hey1 knockdown blocked the osteoclastogenesis inhibition activities of Babam2 (Fig. 8C-D). These results demonstrate that the effects of Babam2 on osteoclastogenesis are dependent on Hey1. Previous studies have indicated that Hey1 could bind the promoter regions of the Nfatc1 to inhibit the transcriptions of Nfatc1. Interestingly, we find that modulating Babam2 expression could significantly influence the expression levels of Nfatc1 in osteoclasts (Fig 2I-J, Fig 3I-J, Fig. S2A, and Fig. S4). Moreover, Q-PCR sassy results showed that silencing Hey1 nearly completely rescued the expression of Nfatc1 in Babam2-overexpressed osteoclasts (Fig. 8E). Based on these findings, we investigated whether Babam2 could cooperate with Hey1 to inhibit the transcriptions of Nfatc1. We assayed the Nfatc1 promoter activities through luciferase assay, we found that overexpression of Babam2 or Hey1 greatly inhibited the Nfatc1 promoter activities. Moreover, the co-overexpression of Babam2 and Hey1 more drastically inhibited the Nfatc1 promoter activities (Fig. 8F). In addition, to test the possibility that Babam2 indirectly regulates Nfatc1 transcription, we analyzed the proximal RANKL signaling in preosteoclasts derived from Babam2-TG mice and WT littermates. The results showed that there was no significant influence of Babam2 overexpression on the activation of the proximal RANKL signaling including NFκB, MAPK, and AKT signaling pathways (Fig. S4A-B). Taken together, these results support the notion that Babam2 cooperates with Hey1 to inhibit Nfatc1 transcription.

## Discussion

Osteoclasts are the sole bone-resorbing cell in the body 36. They are responsible for the removal of damaged bone tissue and then initiate the process of bone remodeling, which is essential for the maintenance of bone homeostasis 37, 38. Under physiological conditions, the formation of osteoclasts is strictly regulated to avoid the excessive activation of bone resorption 1, 39. However, a variety of pathological factors, such as estrogen deficiency, inflammation, glucocorticoid stimulation, tumor invasion, and bone unloading, could stimulate the formation of osteoclasts, leading to the excessive activation of bone resorption, and then lead to the occurrence of a variety of bone diseases 40-42. While antiresorptive bisphosphonate drugs are effective in blocking bone resorptions, however, most have limitations such as destruction of normal bone remodeling and potentially severe side effects including osteonecrosis of the jaw and atypical femoral fractures 43-45. Therefore, deepening the understanding of the regulatory mechanism of osteoclast formation is essential for the development of novel strategies to combat osteoclast-related bone diseases.

Babam2 is an evolutionarily conserved stress-modulating gene, which is an important component of intracellular BRCA1-A and BRISC protein complex, and participates in diverse cellular processes such as DNA damage repair, apoptosis, cell cycle, and so on 46, 47. Babam2 was highly expressed in bone tissue, while its molecular functions and mechanism of action in bone metabolism are still lacking. In this study, we explored the functions of Babam2 in osteoclastogenesis systemically. Firstly, through in vitro gene loss-of-function or gain-of-function studies, we demonstrate that knockdown Babam2 expression significantly accelerated the osteoclast differentiation, conversely, Babam2 overexpression greatly blocked the osteoclast formations. Then, we constructed Babam2-transgenic mice to verify the effects of Babam2 on osteoclastogenesis and bone metabolism. We find that the osteoclast number was significantly decreased and the bone mass was greatly increased in Babam2-transgenic mice. Thus, our results established the essential role of Babam2 on osteoclast formation.

Inflammatory conditions with increasing inflammatory cytokine levels such as TNF-α and IL-6 induce pathological osteoclast formation, causing excessive bone resorption 36, 48. In this study, we constructed a LPS-induced osteolytic model that mimics septic arthritis 8, 12 to validate if Babam2 overexpression could prevent pathological bone resorption. We find that Babam2 transgenic mice are protected from LPS-induced calvarial bone lesions and these results indicate that overexpression of Babam2 is a potential therapeutic approach against pathologic excessive bone resorption.

Osteoclast differentiation is tightly regulated by the sequential expression of multiple hub transcription factors, including PU.1, c-Fos, Mitf, Nfatc1, Cited2, and so on 49. Of these transcription factors, Nfatc1 is the master modulator indispensable for osteoclast differentiation and bone resorption 50. Cellular Nfatc1 levels could be regulated by diverse mechanisms from transcription level to post-transcriptional level 21. For example, transcription factors c-Fos and NF-κB components p50 and p65 are essential for the early induction of Nfatc1 during osteoclast differentiation 51, 52. MicroRNA-124 and MicroRNA-193-3p could direct targeting Nfatc1 to inhibition of osteoclastogenesis 53, 54. The Notch signaling pathway has important functions in regulating skeletal development and bone remodeling 55. Notch1 activation on osteoclast precursors could inhibit osteoclastogenesis 56. The deficiency of Notch cofactor Rbpjκ leads to enhanced osteoclast formation, indicating that Rbpjκ is a negative regulator of osteoclast formation 57. Moreover, Hey1 a target gene of Notch/Rbpjk signaling could directly bind to the Nfatc1 promoter to suppress the transcription of Nfatc1 58. Our results indicate mRNA expression levels of Nfatc1 in osteoclast could be regulated by Babam2. Interestingly, our Co-IP-MS assay identified that Hey1 was a strong binding partner of Babam2. Moreover, we find that the inhibitory effects of Babam2 on osteoclastogenesis were largely dependent on the presence of Hey1, as silencing Hey1 nearly completely diminished the anti-osteoclast activity and rescued the expression of Nfatc1 in Babam2 overexpressed osteoclasts. Furthermore, through promoter activity luciferase assay, we show that Babam2 cooperates with Hey1 to inhibit Nfatc1 transcription. Therefore, in this study, we identify a novel cellular regulatory mechanism for Nfatc1 transcription.

In this study, we find that Babam2 expression was diminished in bone tissues from OVX. However, to the best of our knowledge, the regulatory mechanism of Babam2 expression is still unknown. Therefore, it is of great importance to explore the signaling pathways which regulate the Babam2 expression in bone cells, for example, the estrogen signaling pathways.

In conclusion, our study elucidated the essentially negative role of Babam2 in osteoclastogenesis through both loss-of-function and gain-of-function strategies, suggesting that Babam2 was a potential novel therapeutic target to combat excessive bone resorption-related bone diseases, such as osteoporosis.

## Supplementary Material

Supplementary figures.

## Abbreviations

Babam2: BRISC and BRCA1-A complex member 2
Nfatc1: Nuclear factor of activated T cells, cytoplasmic 1
M-CSF: Macrophage colony-stimulating factor
RANKL: Receptor activator of nuclear factor-κB ligand
BMMs: Bone marrow-derived macrophages
Q-PCR: Quantitative real-time PCR
OVX: Ovariectomy
Ctsk: Cathepsin K
Trap: Tartrate-resistant acid phosphatase
Dcstamp: Dendrocyte expressed seven transmembrane protein
Mmp9: Matrix metalloproteinases 9
