# Supplementary material for: Babam2 negatively regulates osteoclastogenesis by interacting with Hey1 to inhibit Nfatc1 transcription
Source: Int J Biol Sci. 2022 Jul 11;18(11):4482–96. doi: 10.7150/ijbs.72487 (PMC9295054; doi:10.7150/ijbs.72487)
Supplement: Supplementary file 1 — Supplementary figures. [file ijbsv18p4482s1.pdf]

Supplementary materials

Babam2 Negatively Regulates Osteoclastogenesis By Interacting With Hey1 To Inhibit Nfatc1 Transcription

Jin et al.

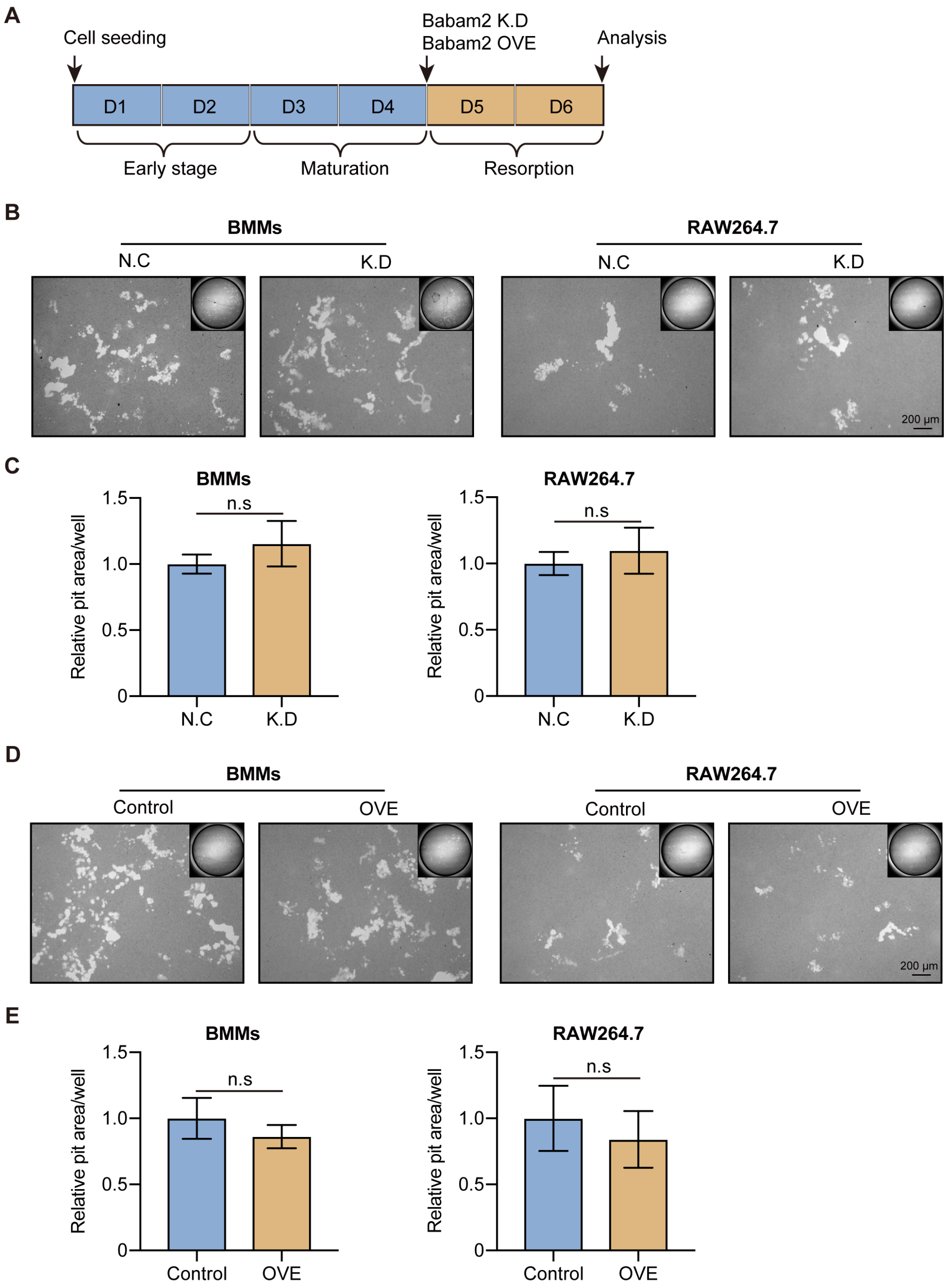

Figure S1. Babam2 did not directly affect osteoclast resorption activity. (A) Schematic diagram illustrating the experimental design. (B) Representative images of the osteoclast resorption pits formed by the control and Babam2 knockdown osteoclasts in Corning Osteo Assay Surface Plate. (C) Quantification of the relative osteoclast resorption area per well (n=3). (D) Representative images of the osteoclast resorption pits formed by the control and Babam2 overexpressed osteoclasts in Corning Osteo Assay Surface Plate. (E) Quantification of the relative osteoclast resorption area per well (n=3). Data are presented as the means ± S.D. n.s, not significant.

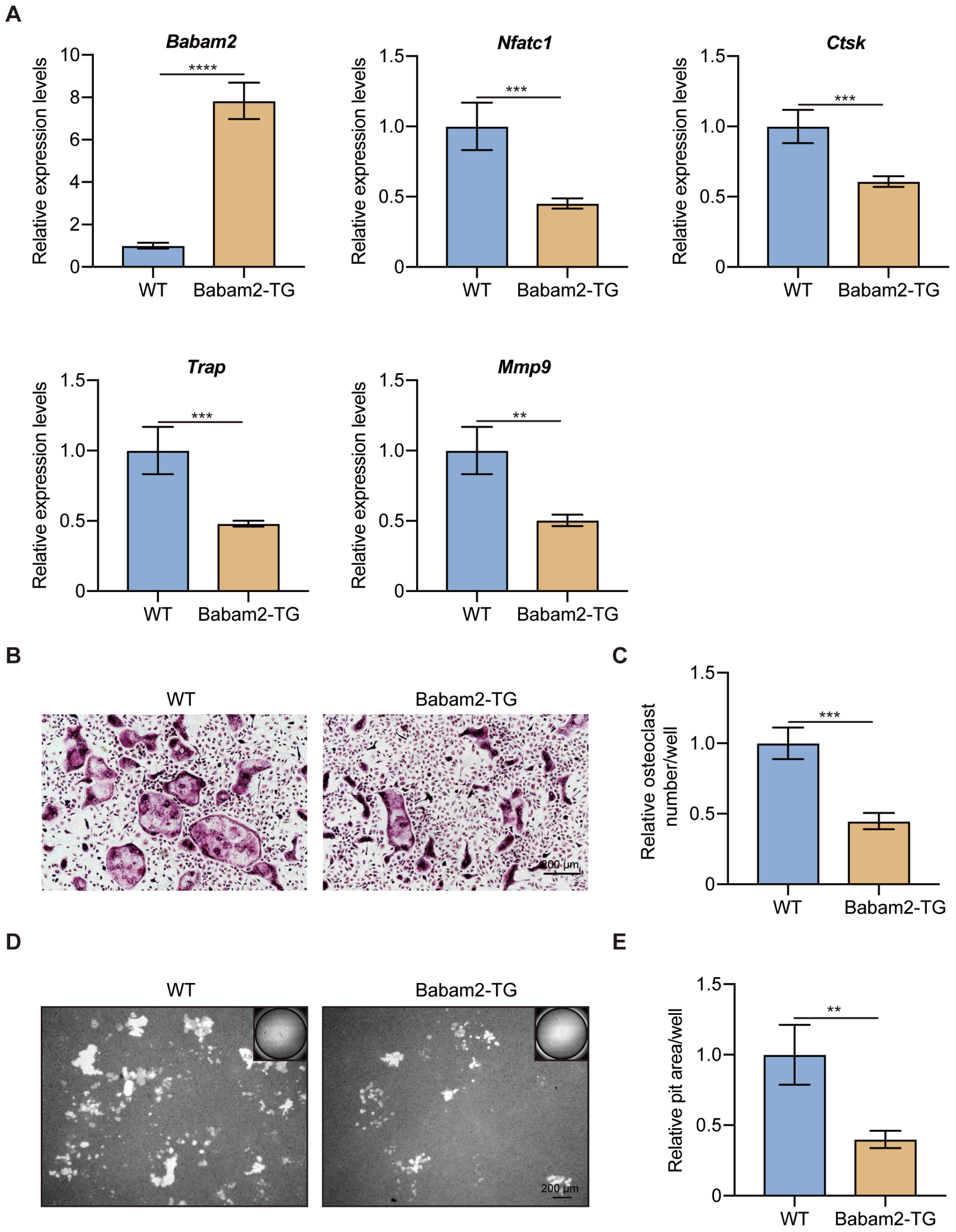

Figure S2. Babam2 inhibits osteoclastogenesis. (A) Q-PCR analysis of *Babam2*, *Nfatc1*, *Ctsk*, *Trap,* and *Mmp9* mRNA expression levels of WT and Babam2-TG mice-derived osteoclasts (n=4). (B) Representative TRAP staining images and (C) quantification of relative osteoclast number per well of the WT and Babam2-TG mice-derived osteoclasts (n=4). (D) Representative images of the osteoclast resorption pits formed in Corning Osteo Assay Surface Plate by the WT and Babam2-TG mice-derived osteoclasts and (E) quantification of relative pit area per well (n=4). Data are presented as the means ± S.D. **P<0.01, ***P<0.001, ****P<0.0001.

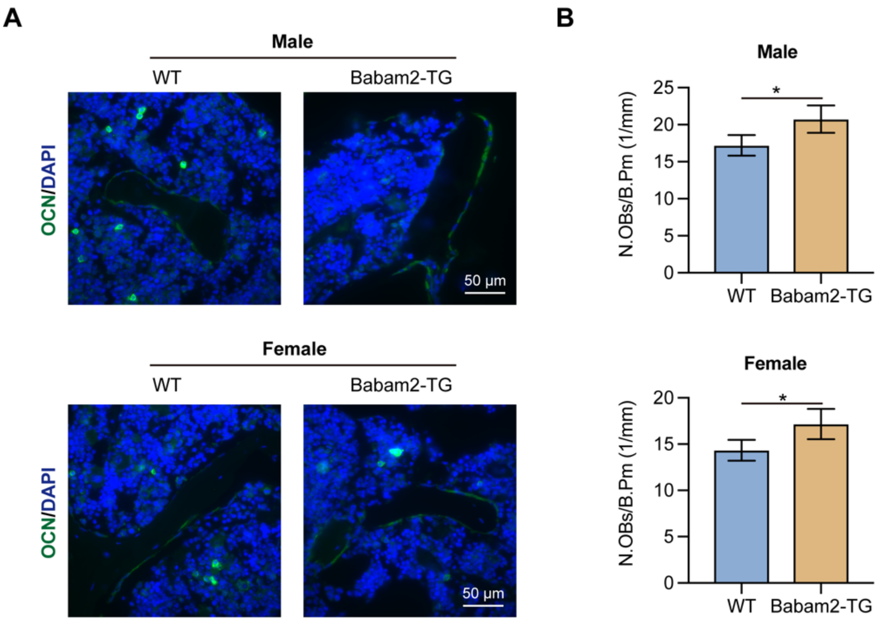

Figure S3. Babam2 overexpression increases osteoblast number in mice. (A) Representative OCN immunofluorescence staining images of the femurs from 3-month-old WT and Babam2-TG mice. (B) Quantification of the number of OCN-positive osteoblasts (N.OBs) per bone perimeter (B.Pm) (n=4). Data are presented as the means ± S.D. **P*<0.05.

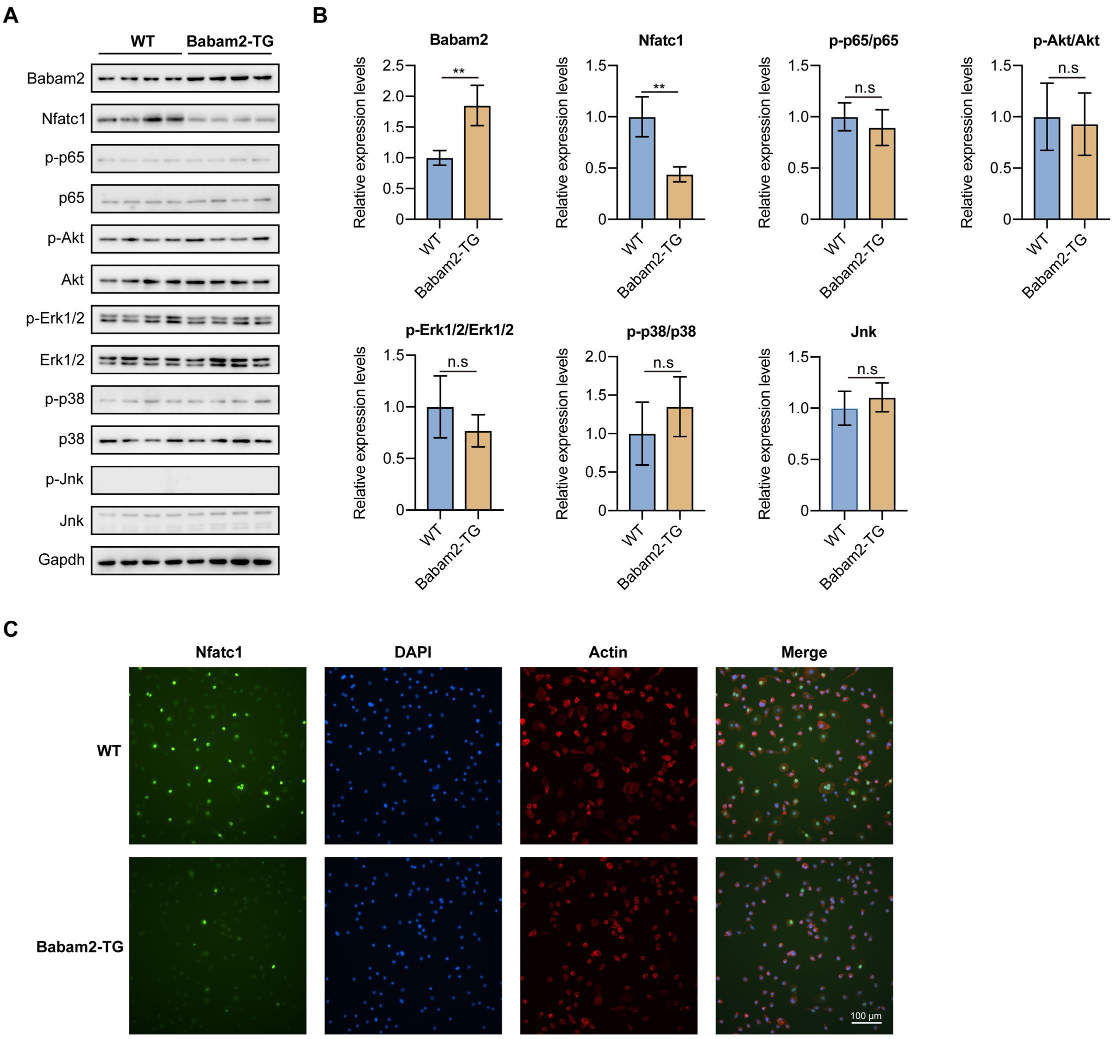

Figure S4. Babam2 did not influence proximal RANKL signaling. BMMs derived from Babam2-TG mice and WT littermates were treated with M-SCF+RANKL for 24 h to generate preosteoclasts and used for western-blot assay or immunofluorescence assay. (A) Western-blot analysis of Babam2, Nfatc1, Gapdh, and key proteins of proximal RANKL signaling including NFκB, MAPK, and AKT signaling pathways. (B) Quantification of protein intensity (n=4). (C) Representative Nfatc1 immunofluorescence staining images of the preosteoclasts derived from Babam2-TG mice and WT littermates. Data are presented as the means ± S.D. n.s, not significant. *P<0.01.
